# Innovation and organizational performance: A perspective among Chinese enterprises

**DOI:** 10.3389/fpsyg.2022.927617

**Published:** 2022-11-07

**Authors:** Chengpeng Zhu, Adubofour Isaac, Nkrumah Nana Kwame Edmund

**Affiliations:** ^1^School of Finance and Economics, Jiangsu University, Zhenjiang, China; ^2^School of Management, Jiangsu University, Zhenjiang, China

**Keywords:** innovation, enterprise performance, moderating role, investor sentiment, Chinese enterprises

## Abstract

The influence of innovation on the performance of Chinese enterprises still remains inconclusive in the literature of innovation management. The aim of this research therefore is to examine the link between innovation and performance of Chinese enterprises, and explore the influence of sentiment expressed by investors in this relationship. The data for our study are drawn from 3,500 Chinese listed firms, operating within the periods, 2009–2017. Panel autoregressive models (fixed and random effects) are employed in our empirical analyses. We further performed Hausman tests in order to ascertain which of the models is more suitable for our dataset. Results from the analysis show that innovation significantly influences the performance of Chinese enterprises and it is moderated by sentiment expressed by investors. Specifically, it is found that Chinese enterprises tend to be innovative as feedback to sentiment expressed by investors and this consequently results in higher performance.

## Introduction

Firms are increasingly resorting to the acquisition of new ideas through the investment in innovation so as to boost their output (Grigoriou and Rothaermel, 2017; Mooi et al., 2020). Whereas some scholars contend that innovation’s influence on firm output is material only at the initial stage (Ahu, 2015; Raphael et al., 2018), others hold that its influence is substantial at every stage of the firm (He, 2019). Despite these varied arguments, extant research has traditionally established that there exists a link between innovation and enterprise output (Shi et al., 2020; Shuiliu et al., 2022). The quest for innovation in corporate activities is said to be market-driven and considered as precondition for survival, sustainability, and growth (Backman et al., 2017; Nuria et al., 2018). Firms embrace innovation in their operational strategies in order to remain in business and maximize profit (Cao and Wang, 2020; Guo et al., 2020). By so doing, quality service delivery is enhanced (Alam et al., 2019; Syed et al., 2021a) as well as the ability to enter into new markets (Ming et al., 2012; Pegah and Peter, 2018). Syed et al., 2021b emphasized the need for technological innovation in the provision of quality services, thereby reducing the negative impact of production on the environment. Innovation is also considered a major factor toward improving firms’ competitiveness (Syed and Yu, 2020), and economic performance (Syed A.R.K. et al., 2020). Extant studies demonstrate the need for innovation in order for firms to fully recover from economic shocks (Francesco C. et al., 2020; Mohammed-Ali et al., 2021) resulting from the effects of a global pandemic (COVID-19), where the supply chain is affected negatively (Elbaz and Salomée, 2021), and makes it difficult for firms in productive sectors to produce at full capacity (Zuari et al., 2021).

Our study argues that the link between innovation and the performance of Chinese enterprises takes an inverted U-shaped pattern. The study underscores that for a firm to continually sustain its market value, it is imperative to strategically obtain an alignment between its objectives and the investment made in innovation. This is because, with rising levels of competition in the business environment, innovation has become a necessity for firms’ competitive advantage (He, 2019; Syed and Yu, 2020). Previous related studies have argued that aligning business objectives with innovation investment plans facilitates an easy execution of innovative ideas (Ming et al., 2012; Li and Wang, 2016; Zhongju et al., 2021). Innovation is therefore considered a major factor that influences firm output and also enables firms to withstand shocks from external business environment (Alex, 2019). Syed et al., 2021b highlighted that firms with high technological capacities in their production processes were able to withstand the ravages of the global pandemic (COVID-19). Moreover, our study contends that innovation still remains “the golden coin” in the global market, hence the decision by the second largest economy in the world (China) to persistently increase her desire for its investment (Asunka et al., 2020; Jia et al., 2021). Thus, China’s investment in R&D resources has witnessed a continuous rise over the last decade.

Notwithstanding the position of extant knowledge on the role of innovation in the performance of firms (Grigorios et al., 2019; Nathan and Rosso, 2022), findings regarding the relationship between innovation and output of Chinese enterprises are still inconclusive (Chiva et al., 2014; Piening and Salge, 2015; Yuefang et al., 2020; Chao et al., 2022; Shuiliu et al., 2022) and more remain to be understood about what triggers firms’ decision to be innovative. What has not been established clearly in literature is the impact of the innovative efforts of Chinese enterprises on their outputs. Whereas some research scholars posit that the innovative efforts of Chinese enterprises have a significant influence on their productivity (Shi et al., 2020; Yuefang et al., 2020; Zhongju et al., 2021), others could not establish the same in their findings (Jiancheng and Nan, 2003; Chao et al., 2022). By responding to the identified shortcoming of inconclusiveness in existing knowledge, our study seeks to investigate the link between the innovative efforts of Chinese firms and their performance, and further delve into identifying the moderating role of investor sentiment in the examined link. Specifically, our research seeks to unravel how the decision by Chinese enterprises operating in China to invest in innovation is triggered and its resultant effect on their financial performance. In order to fill these research gaps, our study responds to the following research concerns; what is the link between innovation and performance of Chinese firms and what role does sentiment of investors play in the examined link? To answer these research questions, innovation-performance relationship analysis is performed, where the capabilities of firms to efficiently manage R&D inputs to obtain specific innovative objectives (Jeff et al., 2020; Yuan et al., 2021) and the influence of sentiment expressed by investors in the innovation-performance relationship are analyzed (Yi and Yang, 2018; David et al., 2019).

In the quest to achieving this aim empirically, the arguments made in the study are tested on a panel of 3,500 Chinese firms, with 31,501 firm-year observations, over the period, 2009–2017. The study’s empirical results provide evidence in support of our argued contentions, suggesting that innovation influences the performance of Chinese firms positively, and it is enhanced by sentiment expressed by investors. However, beyond a certain limit, innovation’s influence on enterprise performance declines, indicating the inverted U-shaped pattern. This study makes theoretical and empirical contributions to the current knowledge in innovation management. Theoretically, our research provides a more nuanced understanding about how the decision by firms to invest in innovation is triggered and the effect on productivity. Research aimed at examining how sentiment of investors enhances the relationship between enterprise innovation and performance is distinct from others to the best of our knowledge. Our study contributes to the scholarly debate on the implications of innovation on the performance of firms (Yuefang et al., 2020; Mohammed-Ali et al., 2021; Zhongju et al., 2021; Shuiliu et al., 2022), and sheds light on factors that moderate the innovation-performance relationship. Empirically, this research improves our understanding of how Chinese enterprises can strengthen their production capabilities by focusing on obtaining firm innovation efficiency rather than placing much emphasis on new product introduction.

Corporate innovation is usually designed as feedback to address the concerns of customers or consumers. As suggested by previous studies, firms plan their innovation strategies to correspond with the preference of customers so as to acquire positive sentiment (Daniel, 2016; Chen et al., 2019). They obtain an increase in revenue in their quest to respond to the opinions of investors (He, 2019). Although previous works have attempted examining innovation’s impact on firm performance (Jen and Scott, 2017), this study expands the boundary of the few existing literature on corporate innovation and performance as it seeks to further investigate the moderating role of investor sentiment in the examined relationship between enterprise innovation and performance, empirically.

The remained sections of the paper are organized as follows: the next section reviews related literature in order to build the conceptual framework of our study. The following section captures our research methodology and data used. It then concludes with a presentation of our major findings, followed by discussing the implications of the study for theory and practice, as well as limitations and future studies.

## Literature review

Notwithstanding that there is knowledge about domestic firms in China employing innovation in their operations in the quest to maximizing output (Yearbook, 2021), knowledge regarding the impact of these innovative efforts on output is not definitive in literature, as well as what triggers the innovation decision. In an attempt to address these, past related works on the relationship between innovation and enterprise performance, as well as the influence of investor sentiment, are reviewed.

### Innovation and enterprise performance

Innovation accounts for a significant portion of investments and changes in business operations (Schumpeter, 1934). Keynes (1936) alluded to the behavioral dynamics of consumers in his “General Theory” and argued that this makes investment outcomes subject to the outmost probability. This probabilistic nature of investment outcomes is said to be a disincentive to innovation investment by firms since most investments made by firms are predicated on expected returns. Scholars in the area of innovation management have however posited that acquiring new ideas is significant for achieving enterprise competitive advantage in modern corporate activities (Jingtao et al., 2021; Sheshadri et al., 2021), hence the need for innovation in firm activities. In this study, research expense of Chinese firms is used as a measure of innovation. The creation and development of new ideas in corporate activities is increasingly gaining much prominence due to its impact on performance (Chen et al., 2020). Studies that capture the significance of innovation in enterprise activities gained much attention after the seminar by Griliches (1979) and Mario et al. (2018). Innovation management researchers have since then advanced the scholarly discourse on the link between innovation and its associated effect on organizational performance (Li and Wang, 2016; Jeff et al., 2020; Chao et al., 2022; Gantert et al., 2022). China is recognized globally for its enormous production capabilities (Hyejin et al., 2018; David et al., 2019; Mingshan et al., 2019; Yongtao et al., 2022); hence studies that seek to establish how the innovation influences the performance of Chinese firms are imperative in current times (Diéguez-Soto et al., 2019; He, 2019). Also, the inconclusiveness of earlier findings (Yuefang et al., 2020) makes this current study necessary.

Corporate innovation basically aims at achieving three objectives, these are: the development of new ideas, combining existing ideas in diverse ways, and improving upon ideas acquired from elsewhere. Achieving these objectives would lead to innovation waves among indigenous enterprises as it is supported by procedures regarding the acquisition of resources (Akinwale, 2018; Cherry et al., 2020; Alexandra et al., 2021). Belhadi et al. (2021) argued that the generation of new ideas remains a critical component of knowledge diffusion among domestic enterprises, in the attempt to creating innovation waves. Domestic businesses can benefit significantly from innovation if there are mass innovation efforts domestically (Belhadi et al., 2021). This is because the generation and development of indigenous knowledge as well as the building of local capacities augment the creation of mass innovation waves for enterprise performance (Teresa et al., 2017; Wan et al., 2021) and the two cannot be delinked. Verhagen et al. (2021) posited that building on previously acquired knowledge is necessary for the general growth of firm innovation and its subsequent effect on productivity. Syed et al. (2022a,b) highlighted that technological innovation enhances the provision of quality services. These arguments therefore enforce the position that firms embrace innovation with the intent to improve output.

Moreover, endogenous growth theory postulates that increased productivity can be attributed directly to a rise in the level of innovation. The theory asserts that higher performance is achieved when firms strategize internally rather than depending on external factors. This suggests that innovation influences organizational productivity when it is incorporated in firm’s operational plans (Aboramadan et al., 2020; Avunduk et al., 2021; Jack et al., 2021), and it can be further enhanced when the culture of innovation is developed (Asunka et al., 2020; Yuan et al., 2021). The theory further asserts that benefits derived from innovation transcends from firm level to the growth of the general economy. It can therefore be argued that adopting innovative ways in organizational activities enhances economic growth (Syed A.R.K. et al., 2020). This is due to the fact that growth in companies resulting from corporate innovation leads to the expansion of the economy through employment and revenues from taxes (Chen and Ibhagui, 2019; Asunka et al., 2020; Jianmin and Li, 2020). It is suggestive that efficient growth of a country’s economy is linked to its ability to acquire and implement new technologies (Metcalfe and Ramlogan, 2008; Syed et al., 2021a; Wan et al., 2021).

Besides, extant knowledge on this subject posits that commitment to innovation is a key factor for determining the future existence of firms (Sheshadri et al., 2021). Although some critics on the subject have denied the role of innovation in the performance of firms (Daniel and Raquel, 2011; Qian et al., 2021), it still remains an undeniable fact that innovation contributes significantly to firm productivity and the growth of a country (Syed and Dong, 2017; Guo et al., 2020). Cherry et al. (2020) indicated that firms that are in areas that are not advanced technologically can increase their productivity through the generation of fresh knowledge and by increasing their investments in research. It is also an established fact that companies that resort to innovation have a higher propensity to withstand shocks emanating from the economy (Chao et al., 2022; Shuiliu et al., 2022). Research has therefore provided compelling evidence suggestive of the fact that innovation is critical in times of crisis (Jingtao et al., 2021; Syed et al., 2022a,b) and firms that tend to be innovative experience a quick turnaround in their operations. Syed et al. (2021c) also emphasized that the introduction of technological innovation in firm’s operational activities impacts positively on global supply chain practices, which is necessary for firms’ post-pandemic survival.

As captured in the literature above, many studies have been conducted regarding the innovation-performance relationship. However, findings regarding the relationship existing between Chinese enterprise innovation and performance still remain inconclusive. This study seeks to advance the knowledge in literature and further provide clarity empirically on the innovation-performance link among Chinese enterprises by investigating how the innovative efforts of Chinese firms impact their performance.

### Investor sentiment and enterprise performance

Economists have over the years emphasized the influence of sentiment in corporate activities. The notion of a relationship between investor emotion and financial performance emphasizes how the attitude, decision, emotion, and judgment of investors influence the investment decision of firms (Jiang et al., 2019; Angeles et al., 2020). Firms that embrace innovation treat sentiment expressed by investors as feedback, and are incorporated in their investment decisions (Chang and Taylor, 2016; Ding and Ou, 2019). This is because getting feedback from investors informs firms about investors’ sensitivity to market trends, and how they evaluate new product concepts introduced (Candi et al., 2018). Also, firms need feedback in order to strategize and develop investment plans (Hoornaert et al., 2017) so as to increase the satisfaction of customers, given that this would result in increased output consequently (Razavi et al., 2016).

Sentiment is said to be derived when there are deviations from the expectations of a customer, investor, or consumer (Milani, 2017; Yang et al., 2019). This deviation may occur as a result of either excessive optimism or pessimism. Sentiment expressed by investors can affect firm’s equilibrium output (Benhabib et al., 2015), and influence business cycle (Hua et al., 2011; David et al., 2019). Investor sentiment can either be positive or negative. Positive sentiment occurs when investors get satisfied or receive information on potential satisfaction and are willing to praise firms for their business strategies while negative sentiment occurs when there is dissatisfaction by investors (Baker and Wurgler, 2007a,b; Wenping et al., 2018). Sentiment is usually attributed to information received which influences the perception and judgment of investors or customers toward an organization or its product (Yao et al., 2018). Positive sentiment is expected to lead to increased productivity by firms since customers would patronize more of the products or services produced by firms when they have information on satisfaction. Negative sentiment on the other hand reduces firm revenue due to reduced patronage and limited capital support from investors. To this end, it can be argued that the decision by firms to invest in innovation to some extent is likely to be underpinned by the sentiment of investors, which would consequently have a long-term effect on the growth of firms.

Factors influencing companies’ decision to be innovative is an area that needs much attention in the literature of innovation management. Much attention is currently focused on how innovation affects productivity (Ahn et al., 2018; Gil-Alana et al., 2020), but this does not provide enough insight into what causes organizations to be innovative. Since extant knowledge has established a link between innovation and enterprise productivity (Grigorios et al., 2019; Mooi et al., 2020), there is a need to better understand the role of investor sentiment in the decision of Chinese firms to embrace innovation. The argument put forward by our study is that sentiment expressed by investors is likely to explain the examined relationship between innovation and a company’s output. Deducing an idea from the study of Yi and Yang (2018), sentiment expressed by investors about firms can push firms to increase their desire for innovation and consequently result in higher productivity. Scholars have over the years been interested in understanding how feedback received by firms in the form of sentiment expressed by investors affects their operational decisions (Qiulin and Karen, 2019), as well as the development of a new product. Our study investigates how sentiment of investors can influence the link between innovation and performance of indigenous firms in China.

Sentiment expressed by investors regarding an enterprise or its product/service is expected to receive response from firms so as to increase investor optimism (Ding and Ou, 2019). Positive sentiment toward a firm or its product helps to increase revenue as well as the investment in R&D (Cai et al., 2019). Investor sentiment is therefore a critical factor in making innovation decisions. Qiulin and Karen (2019) argued that the perception of investors has great influence on enterprise innovation investment as well as the general growth of enterprises. Companies that give credence to the sentiment of customers tend to improve the quality of service delivery (Axel and Stephan, 2019), and this impacts the financial position of firms in the long run. Li and Wang (2016) observed that the perception of customers influences the relationship that exists between firm’s innovative efforts and performance in the pharmaceutical sector of China.

Firms that regard the opinion of customers in their strategic decisions often experience a rise in total productivity (Hoornaert et al., 2017). Past research work has indicated that investor opinion has long-term impact on the performance of companies (Junyan et al., 2017). A study by Cai et al. (2019) showed that companies deliberately produce in response to the sentiment expressed by investors. This response can have a significant impact on firm revenue. The quest to satisfying the demands of customers so as to obtain positive sentiment has a major impact on the future financial outlook of firms. The decision by firms to obtain favorable opinions from customers influences their investment plans (David and Dayong, 2019). They therefore tend to be innovative so as to satisfy their investors which then leads to higher growth (David et al., 2019).

As observed in the above literature, past studies have attempted to ascertain the link between firm’s innovative efforts and its performance. However, the influence of investor behavior in this link is missing in literature. This study therefore aims at examining the role of investor sentiment in the examined innovation-performance nexus.

## Materials and methods

In order to achieve the objectives of this research, we adopt Wenping et al. (2018) panel autoregressive models (fixed- and random-effect models) to examine the relationship between innovation and performance of Chinese firms, as well as examine the moderating role of investor sentiment in this relationship. We also performed Hausman tests to verify which of the models is suitable for our dataset. The Hausman test provides information on the suitability of the two models, fixed effect and random effect, for our dataset.

### Data and data description

Data for the study are obtained from the annual report of Chinese firms, the ifind database, and the China stock market. It consists of a multi-industry sample of Chinese firms and covers the period 2009–2017. The panel data cover 3,500 Chinese listed enterprises with 31,501 observations, consisting of private and state-owned enterprises. The study’s sample is constructed by drawing data from multiple sources, Data on investor sentiment are obtained from firms’ listed on the stock market of China in the Shanghai and Shenzhen stock exchanges. Also, firm performance and innovation investment data are collected from the ifind database where Chinese firms’ annual R&D investments and performance information are reported. These data are used to construct our panel for the analysis.

#### Dependent variable

Firm performance (FP). The returns made on the assets of firms are captured as the dependent variable for measuring the performance of Chinese firms. It is a widely acknowledged indicator for measuring firm’s financial performance (Kim and Youm, 2017; Mario et al., 2018; Elaine et al., 2020), and it is denoted in this study as $FP_{i,t}$ . Return on assets is a profitability ratio for determining the efficient ways for firms to generate earnings relative to investments made in firms’ operations. It measures the ability of firms to generate profits out of their assets, irrespective of their size. A high ratio signifies greater returns and it shows how well an organization generates profit from its total assets while a low ratio indicates low performance. Investors use ROA to compare different companies in making investment decisions (Koutroumpis et al., 2020).

#### Independent variables

Firm innovation. The yearly R&D expense of indigenous enterprises in China is used as an indicator for determining firm innovativeness. This is a generally accepted indicator by scholars in the field of innovation management (Czarnitzki and Hottenrott, 2011; Wenping et al., 2018) and it is denoted in our study as $R\&D_{i,t}$ . Research and development investments are used to determine the desire of firms for innovation.

##### Investor sentiment

Our analysis aims at examining how sentiment by investors influences the innovation-performance relationship. Monthly stock returns from the stock market of China are used as an indicator for measuring the sentiment of investors (Hua et al., 2011; Francesco A. et al., 2020; Jiangshan et al., 2021) and denoted as $SR_{i,t}$ . An increase in stock returns signifies positive sentiment of investors while reduced returns also indicate negative sentiment from investors. Sentiments are regarded as feedbacks from investors after a product or service is utilized, or the information available to investors upon which decisions and judgments are made. Highly positive sentiment is expected to increase firm revenue while highly negative sentiment should cause firms to be innovative so as to address the dissatisfaction of investors.

The monthly stock returns is mathematically expressed as:


1 $$SR_{t}=\ln P_{t}-\ln P_{t-1}$$

Where $SR_{t}$ signifies stock returns, and $P_{t}$ represents closing price in month *t* which is as well the stock market price.

Investor sentiment proxy measures are considered due to the dynamics of the Chinese stock market. These proxy indicators are used to consider the measurement of investor sentiment.

First, Market Turnover Rate (MTRN). This approach asserts that liquidity can be used as a measure of sentiment (Baker and Stein, 2004). It contends that investors tend to have reduced opinions in the downturn of the stock market and high opinions when the stock market experiences an upsurge. Market liquidity can therefore be measured by the rate of turnover in the market.

Second, Growth Rate of Newly Opened A-Share Accounts (RNOA). As posited by Wu and Han (2007), market enthusiasm will rise when investor optimism is high and this will reflect in the number of accounts opened. This equation is stated as;


2 $$RNOA=\frac{NOA}{TNOA}$$

Where *NOA* signifies newly opened accounts in the current month and *TNOA* also represents the total number of newly opened accounts as at the end of the last month.

Third, Number of Monthly IPO (NMIPO) and its First-Day Return (FIPO). The number of stock offerings and first-day returns can both reflect sentiment expressed by investors (Baker and Wurgler, 2006). A rise in the issuing scale is expected to lead to a corresponding rise in the returns of the first day. So in general, when the sentiment expressed by investors is high, the issuance of new shares is also intensive and vice versa. The number of IPOs refers to the amount of money raised in initial public offerings monthly.

Fourth, Discount of Closed-End Fund (DCEF). It has been highlighted by Lee et al. (1991) that discount rate of close-end funds can be a proxy for measuring investor sentiment. High discount rate of close-end funds leads to lower sentiment by investors. This can be expressed mathematically as;


3 $$DCEF_{t}=\frac{1}{k}\overset{k}{\underset{i=1}{\sum }}\left(\frac{P_{it-NVA_{it}}}{NVA_{it}}\right)$$

Where $P_{it}$ denotes the market price of fund *i* at the end of the month *t*, $NVA_{it}$ also denotes fund’s net value at the end of the month *t*, and *k* represents number of closed-end funds at the end of the month *t*.

#### Control variables

Variables that have the propensity to impact on firm performance are controlled for in our analyses (Wooldridge, 2010; Yongtao et al., 2022).

##### Assets ( $AST_{it}$ )

Firm’s assets can determine its business position. Assets are expected to be beneficial to firm’s operations due to their economic resource nature (Maggina and Angelos, 2012; Nhung et al., 2021). Firms with large number of assets are expected to be translated into higher productivity. This is an important factor for firms in gaining competitive advantage over those with small assets. Therefore, our study included the assets of each firm as a factor that could impact firm performance.

##### Assets growth rate ( $AGR_{it}$ )

The rate of growth of company’s assets determines its performance in the business environment. Firms with higher growth rates would enjoy higher financial growth (Nhung et al., 2021). For this reason, we added this variable in our model as control variable.

##### Number of employees ( $NEM_{it}$ )

Our study used the number of employees in each firm to determine its size. Intuitively, firms with large size are expected to engage in more capital investments than those with small size, and these investments are also expected to reflect in future financial performance of firms. The size of firm can have impact on its performance (Syed D.H. et al., 2020; Feras, 2021), the variable firm size, is therefore included in our model.

##### Operating income ( $OPI_{it}$ )

Performance of firms is dependent upon its income levels. Firms that obtain higher profits are said to have performed higher than those that obtain low profit margins (Jang and Park, 2011; Antonio et al., 2021). High profit signifies efficient use of firm assets. This variable is added to the model as a control variable.

##### Cash flow ( $CF_{it}$ )

A good cash flow can have impact on the performance of firms (Elaine et al., 2020). Firms with good cash flow can increase their financial position (Rahman and Sharma, 2020), and it is also critical for improving the efficiency of firms’ financial decisions. For this reason, cash flow is added in our models as a control variable.

##### Asset liability ratio ( $ALR_{it}$ )

Enterprise assets to liabilities ratio has impact on performance. Firms with higher assets over liabilities would have higher performance (Feras, 2021). The asset-liability ratio determines the capital strength of a company (Hoang et al., 2020) for the acquisition of materials and maintenance of business operations (Yazdanfar and Peter, 2015). This variable is included in our models.

##### Shareholders equity ( $SHEQ_{it}$ )

The equity holding of investors has influence on firm performance (Zandi et al., 2019). Shareholders’ equity defines the modes that firms finance their investment and operations (Jusoh, 2016; Zachary et al., 2019). We therefore controlled for shareholders’ equity.

##### Earnings per share ( $EPS_{it}$ )

This is used to measure the proportion of an enterprise’s net income assigned to each share holding. Companies that allot high earnings per share would attract more investors, which will have impact on their financial position in the long run (Sathasivam, 2014). Thus we included EPS in our models.

##### Enterprise category ( $ENT_{i}$ )

The performance of firm may vary depending on the category of firm, whether large or small. Firms operating in high technological industries are likely to make more investments in their operations (Wenping et al., 2018), and that can impact their future performance. A categorical variable, $ENT_{i}$ , is therefore constructed to control for variations among enterprises.

##### Year dummy ( $YEAR_{t}$ )

Finally, innovation’s influence on firm performance can change over time. Year dummy variables are therefore included in our estimation models.

### Empirical strategy

Econometric models are adopted in the quest to empirically examine the link between innovation and the performance of Chinese enterprises as well as examine how sentiment expressed by investors enhances this relationship. The study adopted the panel AR (Autoregressive) process with lagged variables for its empirical analysis. Both fixed-effect and random-effect model analyses are applied in order to prevent anti-conservative standard errors, assuming that random intercepts are Normally distributed when they are not (Bell et al., 2019), thereby introducing modest biases. Also, random-effect models allow for more general conclusions and allow for more accurate inferences (Wenping et al., 2018). As noted earlier, we constructed a panel of 3,500 firms with 31,501 observations to empirically assess our research objectives.

The first stage of the analyses is to verify the relationship existing between the innovative efforts of Chinese firms and the resultant effect on their performance. Firm performance is regressed on innovation so as to verify the link existing between innovation and performance. Other relevant variables that can foster higher firm performance are included, as well as an error term. The equation is stated as:


(4) $$\log FP_{i,t}=\alpha_{0}+\alpha_{1}\log\;\left(R\&D_{i,t-1}\right)+\beta Z_{i,t-1}+\varepsilon_{i,t}$$

Where return on assets is an indicator for measuring firm performance, R&D denotes research and development expense of firms (an indicator for mearing innovation), *β* is a coefficient, and *Ɛ* is the error term. Subscript denotes measures across firm (*i*) and year (*t*).

The second stage of our analyses is to investigate the impact of sentiment on the performance of Chinese enterprises. This is intended to investigate the long-run effect of sentiment on firm’s output. The model is therefore estimated as follows:


(5) $$\log FP_{i,t}=\alpha_{0}+\alpha_{1}SR_{i,t-1}+\beta Z_{i,t-1}+\varepsilon_{i,t}$$

The third stage is an analysis on the interactive effect of investor sentiment in the link between Chinese enterprise innovation and performance. The estimated moderating model is presented as:


(6) $$\begin{array}{c} \log FP_{i,t}=\gamma_{0}+\gamma_{1}\log\left(R\&D_{i,t-1}\right)+\gamma_{2}SR_{i,t-1}\\ +\gamma_{3}\log\left(R\&D_{i,t-1}\right)*\left(SR_{i,t-1}\right)+\beta Z_{i,t-1}+\varepsilon_{i,t} \end{array}$$

Where SENT denotes investor sentiment.


(7) $$\begin{array}{c} Z=\beta_{1}\log OPI_{it-1}+\beta_{2}\log AST_{i,t-1}+\beta_{3}\log NEM_{i,t-1}\\ +\beta_{4}\log ALR_{i,t-1}+\beta_{5}DPS_{i,t-1}+\beta_{6}SHEQ_{i,t-1}\\ +\beta_{7}\log CF_{i,t-1}+\beta_{8}EPS_{i,t-1}+\beta_{9}\log AGR_{i,t-1}\\ +\beta_{10}ENT_{i}+\beta_{11}YEAR_{t} \end{array}$$

Variable *Z* captures an extensive set of factors with potential impact on the performance of Chinese enterprises, and also minimizes any potential endogeneity threat. Firm-size-related variables are transformed using logarithm as a result of the variation in the size of firms.

### Statistical analysis

Table 1 captures the descriptive statistics for the studied variables. In this statistics, information on the mean, standard deviation, and other relevant parameters are presented. Table 2 presents the correlation among the variables. Most of the bivariate correlations reported are below the recommended 0.70 threshold (Robinson and Schumacker, 2009), and a majority of the correlations presented are statistically significant at a 1% level.

**Table 1:** Descriptive statistics.

Variable	Obs.	Mean	SD	Min.	25th %	75th %	Max
*FP_it_* (log transformed)	26,884	1.970	0.879	−9.210	1.456	2.566	7.696
*R&D_it_* (log transformed)	21,192	17.286	1.541	6.685	16.441	18.172	23.646
*SR_it_*	31,501	600760.400	200.842	600052.500	600,845	600,845	600,845
*OPI_it_* (log transformed)	20,885	2.854	1.213	−5.240	2.276	3.583	10.512
*AST_it_* (log transformed)	29,232	21.672	1.620	11.348	20.606	21.511	30.892
*NEM_it_* (log transformed)	28,304	7.389	1.386	1.099	6.506	8.214	13.223
*AGR_it_* (log transformed)	23,489	2.842	1.341	−5.521	2.130	3.612	13.065
*ALR_it_* (log transformed)	29,232	3.640	0.659	−1.757	3.311	4.101	9.535
*CF_it_* (log transformed)	28,963	2.718	0.839	−5.150	2.250	3.286	4.605
*SHEQ_it_*	24,683	36.131	16.060	8.520	23.550	46.940	79.380
*DPS_it_*	16,495	0.164	0.232	0.010	0.050	0.200	1.000
*EPS_it_*	26,731	0.495	0.779	−0.857	0.130	0.680	2.300
*ENT_it_*	31,501	Categorical variable. There are 3,500 firms					
*YEAR_t_*	31,501	Category variable. There are 9 years					

**Table 2:** Correlation between variables.

Variable	*FP_it_*	*R&D_it_*	*SR_it_*	*OPI_it_*	*AST_it_*	*NEM_it_*	*AGR_it_*	*ALR_it_*	*CF_it_*	*SHEQ_it_*	*DPS_it_*	*EPS_it_*
*FP_it_*	1											
*R&D_it_*	−0.07^***^	1										
*SR_it_*	0.18^***^	−0.03^***^	1									
*OPI_it_*	0.13^***^	0.01	0.04^***^	1								
*AST_it_*	−0.33^***^	0.52^***^	−0.24^***^	−0.03^***^	1							
*NEM_it_*	0.14^***^	0.54^***^	−0.14^***^	−0.12^***^	0.73^***^	1						
*AGR_it_*	0.28^***^	−0.03^***^	0.10^***^	0.30^***^	−0.04^***^	−0.08^***^	1					
*ALR_it_*	−0.13^***^	0.11^***^	−0.17^***^	0.05^***^	0.33^***^	0.28^***^	−0.01^***^	1				
*CF_it_*	0.18^***^	0.01	0.14^***^	0.03^***^	−0.16^***^	−0.11^***^	0.19^***^	−0.37^***^	1			
*SHEQ_it_*	0.13^***^	0.04^***^	0.01	−0.07^***^	0.13^***^	0.15^***^	−0.00	0.01	0.03^***^	1		
*DPS_it_*	0.38^***^	0.08^***^	0.01^***^	0.02^**^	0.09^***^	0.12^***^	0.11^***^	−0.12^***^	0.17^***^	0.11^***^	1	
*EPS_it_*	0.43^***^	0.11^***^	0.08^***^	0.09^***^	0.02^***^	0.05^***^	0.23^***^	−0.10^***^	0.17^***^	0.13^*^	0.80^***^	1

^*^p < 0.1; ^**^p < 0.05; ^***^p < 0.01.

## Results

### Regression results

Panel autoregressive analyses were employed in the quest to empirically examine the link between innovation and performance of domestic firms in China, and understand the moderating influence of investor sentiment in this link.

The empirical analyses first examined how domestic enterprise innovative capabilities in China impact performance. The results are reported in Table 3. The results show that innovation has positive influence on the performance of Chinese enterprises, as captured in Model 2 (*β* = 0.059, *p* < 0.01), suggesting that innovative efforts by Chinese firms have impact on their performance. This is consistent with previous findings by Syed et al. (2021d) and Guo et al. (2020), where it was established that innovation fosters enterprise productivity.

**Table 3:** Estimation results for the impact of innovation.

Variable	Firm Performance (ROA)	
	Random-effect, Model 1	Fixed-effect, Model 2
$R\&D_{i,t-1}$	0.009 (0.006)	0.059^***^ (0.010)
$OPI_{i,t-1}$	0.045^***^ (0.004)	0.049^***^ (0.005)
$AGR_{i,t-1}$	0.084^***^ (0.004)	0.086^***^ (0.004)
$AST_{i,t-1}$	−0.191^***^ (0.009)	−0.265^***^ (0.016)
$NEM_{i,t-1}$	0.094^***^ (0.010)	0.061^***^ (0.016)
$ALR_{i,t-1}$	−0.091^***^ (0.012)	0.004 (0.016)
$CF_{i,t-1}$	−0.035^***^ (0.009)	−0.011 (0.011)
$SHEQ_{i,t-1}$	0.005^***^ (0.000)	0.006^***^ (0.001)
$DPS_{i,t-1}$	−0.389^***^ (0.036)	−0.523^***^ (0.042)
$EPS_{i,t-1}$	0.661^***^ (0.015)	0.697^***^ (0.018)
$ENT_{i}$	Categorical variable	_
$YEAR_{t}$	_	_
Cons	4.825^***^ (0.154)	5.411^***^ (0.256)
$R^{2}$ : Within	0.786	0.795
$R^{2}$ : Between	0.719	0.668
$R^{2}$ : Overall	0.668	0.635

^***^p < 0.01; ^**^p < 0.05. According to the Hausman test, $\chi^{2}$ (10) = 289.90, *p* < 0.05. Hence, the fixed-effect model fit the data set better than random-effect model.

Also, the study examined the direct relationship between investor sentiment and firm performance. It is observed that investor sentiment has no direct relationship with Chinese firms’ performance (*β* = 0.759, *p* > 0.01, in Model 4). This is presented in Table 4. However, it has been previously highlighted by Razavi et al. (2016) and Milani (2017) that investor sentiment has direct influence on firms’ innovative decisions.

**Table 4:** Estimation results for the impact of investor sentiment.

	Organizational performance (ROA)	
	Random-effect, Model 3	Fixed-effect, Model 4
$SR_{i,t-1}$	−0.000 (0.000)	0.759 (0.241)
$OPI_{i,t-1}$	0.042^***^ (0.004)	0.058^***^ (0.004)
$GRW_{i,t-1}$	0.078^***^ (0.003)	0.109^***^ (0.004)
$NEM_{i,t-1}$	−0.180^***^ (0.007)	−0.192^***^ (0.011)
$AST_{i,t-1}$	0.080^***^ (0.008)	0.045^***^ (0.012)
$ALR_{i,t-1}$	−0.095^***^ (0.011)	−0.045^***^ (0.014)
$CF_{i,t-1}$	−0.031^***^ (0.008)	0.051^***^ (0.009)
$SHEQ_{i,t-1}$	0.005^***^ (0.000)	0.015^***^ (0.001)
$DPS_{i,t-1}$	−0.405^****^ (0.033)	0.621^***^ (0.030)
$EPS_{i,t-1}$	0.679^***^ (0.013)	0.717^***^ (0.016)
$ENT_{i}$	Categorical variable	_
$YEAR_{t}$	_	_
Cons	28.680 (24.984)	5.561^***^ (0.239)
$R^{2}$ : Within	0.694	0.751
$R^{2}$ : Between	0.711	0.664
$R^{2}$ : Overall	0.673	0.623

^***^p < 0.01; ^**^p < 0.05. According to the Hausman test, $\chi^{2}$ (10) = 845.83, *p* < 0.05. Hence, the fixed-effect model fit the data set better than random-effect model.

Our study’s final analysis, thus, examining the moderating influence of investor sentiment in the link between innovation and performance of domestic firms in China, established that investor sentiment moderates the innovation-performance relationship. The results are reported in Table 5. It shows that investor sentiment significantly enhances the link between firm innovation and expected output (*β* = 0.000, *p* < 0.001, in Model 6). It is therefore suggestive of the fact that sentiment expressed by investors’ causes firms to be innovative which consequently improves their performance. This conclusion has been previously argued by Junyan et al. (2017), where it was indicated that investor opinions significantly influence firm’s innovativeness and that consequently affects productivity. Ding and Ou (2019) posited that investor sentiment expressed by investors serves as feedback to firms in their innovation decisions and that would affect productivity in the long term.

**Table 5:** Estimation results for moderating role of investor sentiment.

	Organizational performance (ROA)	
	Random-effect, Model 5	Fixed-effect, Model 6
$R\&D_{i,t-1}$	−0.098^***^ (0.005)	−0.161^***^ (0.007)
$SR_{i,t-1}$	0.001^***^ (0.000)	−0.150 (0.284)
$SR_{i,t-1}$ * $R\&D_{i,t-1}$	0.000 (0.000)	0.000^***^ (0.000)
$OPI_{i,t-1}$	0.045^***^ (0.004)	0.049^***^ (0.005)
$GRW_{i,t-1}$	0.084^***^ (0.004)	0.085^***^ (0.004)
$AST_{i,t-1}$	−0.190^***^ (0.009)	−0.253^***^ (0.015)
$NEM_{i,t-1}$	0.096^***^ (0.010)	0.065^***^ (0.016)
$ALR_{i,t-1}$	−0.090^***^ (0.012)	0.005 (0.016)
$CF_{i,t-1}$	−0.035^***^ (0.009)	−0.012 (0.011)
$SHEQ_{i,t-1}$	0.005^***^ (0.000)	0.006^***^ (0.001)
$DPS_{i,t-1}$	−0.389^****^ (0.036)	−0.525^***^ (0.042)
$EPS_{i,t-1}$	0.662^***^ (0.015)	0.670^***^ (0.018)
$ENT_{i}$	Categorical variable	_
$YEAR_{t}$	_	_
Cons	4.855^***^ (0.153)	5.404^***^ (0.256)
$R^{2}$ : Within	0.685	0.694
$R^{2}$ : Between	0.719	0.773
$R^{2}$ : Overall	0.668	0.739

^***^p < 0.01; ^**^p < 0.05. According to the Hausman test, $\chi^{2}$ (12) = 274.68, *p* < 0.05. Hence, the fixed-effect model fit the data set better than random-effect model.

#### Robustness checks

Robustness checks analyses are conducted to verify how consistent our findings are, relating to the link between innovation and the performance of indigenous firms in China. This is done by extracting two samples from the dataset based on the size of firms (Wenping et al., 2018). The top 25% (large firms) as well as the bottom 25% (small firms) indicated by the number of employees are selected and used to re-examine our main findings.

The analysis tested the relationship between firms’ innovative efforts and the impact on performance. The findings confirmed a significant and positive relationship between firm innovation and performance among the largest firms, as reported in Table 6.

**Table 6:** Estimation results for the top one fourth largest firms.

	Organizational performance (ROA)	
	Random-effect, Model 7	Fixed-effect, Model 8
$R\&D_{i,t-1}$	0.009 (0.006)	0.053^***^ (0.009)
$OPI_{i,t-1}$	0.045^***^ (0.004)	0.049^***^ (0.005)
$AGR_{i,t-1}$	0.085^***^ (0.004)	0.085^***^ (0.004)
$AST_{i,t-1}$	−0.192^***^ (0.009)	−0.261^***^ (0.016)
$NEM_{i,t-1}$	0.095^***^ (0.010)	0.063^***^ (0.016)
$ALR_{i,t-1}$	−0.091^***^ (0.012)	0.004 (0.016)
$CF_{i,t-1}$	−0.035^***^ (0.009)	−0.012^***^ (0.011)
$SHEQ_{i,t-1}$	0.005^***^ (0.000)	0.006^***^ (0.001)
$DPS_{i,t-1}$	−0.382^***^ (0.036)	−0.523^***^ (0.042)
$EPS_{i,t-1}$	0.656^***^ (0.015)	0.698^***^ (0.018)
$ENT_{i}$	Categorical variable	_
$YEAR_{t}$	_	_
Cons	4.850^***^ (0.153)	5.413^***^ (0.256)
$R^{2}$ : Within	0.604	0.694
$R^{2}$ : Between	0.638	0.678
$R^{2}$ : Overall	0.618	0.639

^***^p < 0.01; ^**^p < 0.05. According to the Hausman test, $\chi^{2}$ (10) = 280.80, *p* < 0.05. Hence, the fixed-effect model fit the data set better than random-effect model.

The same analysis was carried out for the bottom 25% of firms. The results for the small-sized firms’ category are captured in Table 7. The findings also corroborate our main results. Hence, innovation can be said to significantly influence the output of small firms as well. These results explain the productive nature of firms in China as they advance their innovative efforts (d’Artis and Siliverstovs, 2016; Rupika and Chandan, 2018; Alam et al., 2019).

**Table 7:** Estimation results for the 25% smallest firms.

	Firm performance (ROA)	
	Random-effect, Model 9	Fixed-effect, Model 10
$R\&D_{i,t-1}$	0.013 (0.029)	0.077^***^ (0.061)
$OPI_{i,t-1}$	0.016^***^ (0.028)	0.011^***^ (0.030)
$AGR_{i,t-1}$	−0.012 (0.029)	−0.001 (0.033)
$NEM_{i,t-1}$	0.016^***^ (0.070)	−0.050^***^ (0.049)
$AST_{i,t-1}$	−0.124^**^ (0.048)	−0.177 (0.112)
$ALR_{i,t-1}$	0.061 (0.101)	0.155 (0.121)
$CF_{i,t-1}$	−0.043 (0.065)	−0.126 (0.094)
$SHEQ_{i,t-1}$	0.033 (0.134)	−0.130 (0.218)
$DPS_{i,t-1}$	−0.105 (0.058)	−0.070 (0.068)
$EPS_{i,t-1}$	0.503^***^ (0.064)	0.438^***^ (0.081)
$ENT_{i}$	Categorical variable	_
$YEAR_{t}$	_	_
Cons	4.376	5.545
$R^{2}$ : Within	0.506	0.642
$R^{2}$ : Between	0.549	0.697
$R^{2}$ : Overall	0.520	0.566

^***^p < 0.01; ^**^p < 0.05. According to the Hausman test, $\chi^{2}$ (10) = 100.07, *p* < 0.05. Hence, the fixed-effect model fit the data set better than random-effect model.

## Conclusion and discussion

In this research, we verified the link that exists between innovation and performance of Chinese firms and examined the moderating role of investor sentiment in this link. Despite the general role of innovation in enterprise productivity (Nina and Meluzin, 2016; Hyejin et al., 2018; Jeff et al., 2020; Shi et al., 2020), its impact on the growing productivity of firms in China still remains inconclusive (Yuefang et al., 2020; Zhongju et al., 2021), as well as what influence this link. Specifically, our study underscored that the role investor sentiment plays in the innovation-performance relationship has not been captured properly in both past and current literature. In order to address this gap in literature, we employed panel autoregressive models in our analysis.

The results of the analysis indicate that innovation significantly influences the performance of firms in China, consistent with earlier studies that suggest that the innovative efforts of firms in China are positively associated with their performance (Mingshan et al., 2019; Shuiliu et al., 2022). These findings provide evidence on factors leading to the growing productive pace of China’s industries. Also, it was found that sentiment expressed by investors positively enhances the link between innovation and the performance of Chinese firms. More specifically, positive opinions send a signal to firms that investors are satisfied with their current services and that leads to an expansion of innovative efforts by firms in order to maintain the desired satisfaction. Firms that receive large amount of positive responses often see the need to invest in innovation since its returns can be easily experienced in terms of firm revenue. Firms may also be motivated to pay much attention to innovation when they receive negative responses from investors, especially strongly negative opinions. Companies are expected to align their operational investment decisions with their innovative efforts so as to continuously receive positive responses from investors. This study remains one of the few works to investigate how sentiment expressed by investors can moderate the link between innovation and the performance of Chinese enterprises.

Furthermore, findings from the robustness analyses provide a corroborated evidence for the link between innovation and performance of Chinese enterprises. The results reveal that innovation is significantly associated with the country’s production capabilities (Baesu et al., 2015; Ahn et al., 2018; Yu et al., 2021). These results affirm the consistency of our findings regarding the link between innovation and financial performance of Chinese enterprises.

### Implications for theory

Theoretically, this research contributes to the scholarly discourse regarding innovation and performance of Chinese enterprises operating in China (Cai et al., 2019; Nathan and Rosso, 2022; Shuiliu et al., 2022) by providing evidence on the relationship that exists between innovation and the performance of Chinese enterprises. Previous studies have not fully paid attention to how the innovative effort of firms in China influences their production capabilities. This study provides understanding of how innovation fosters performance of domestic firms in China. Moreover, our study highlights the theoretical boundary of how sentiment expressed by investors moderates the firm innovation-performance relationship. Scholars have not explored the influence that investor sentiment has on the productivity of companies. In the context of our study, the sentiment expressed by investors in China is found to have influence on firm’s level of innovativeness which consequently affects performance. More specifically, the results of our study will be useful in providing adequate information to firms that do not incorporate innovation in their operations since it has a significant influence on financial performance.

### Practical implications

The study also provides contribution to practitioners in addition to its contribution to current literature. The study’s result reveal a significant association between innovation and firm performance, and this has major implications for firm managers. First, it provides insight on how managers can strengthen firms’ competitive position by advancing their innovative efforts. Our study provides evidence to managers of firms in China who seek to make innovation investment decisions, that such investments are essential for firm survival in a highly competitive business environment.

The analysis on investor sentiment provides an intuitive understanding of investor behavior and responses. This can enable firm managers to pay attention to the dynamics of investor sentiment as antecedent for operational analysis. The culture among Chinese is ordinarily considered more collectivist, hence, more adaptable to the “word-of-mouth effect” (Hong and Davison, 2010). The sentiment analysis with the focus on Chinese investors would therefore be useful for firms seeking to obtain understanding about the nature of investors in China. The study’s result provides information to managers on the need to regard the sentiment of investors since it showed a significant influence on the output of domestic enterprises in China. It is also important for the government of China to support the investment in innovation by providing firms with innovation incentives since its impact is felt on the general economy through the creation of employment.

### Limitations and future research

This research has some limitations that can encourage further studies. First, data for this research are collected on Chinese domestic firms; hence, care should be taken when generalizing the results for firms in other countries. Future research is encouraged to extend the dataset to other countries within the continent for the results to reflect a broader context for generalizability. Second, it was observed that some of the studied variables had incomplete data. Further studies should therefore analyze the complete dataset if available. Although investor sentiment showed to enhance the link between enterprise innovative capabilities and financial output, other factors of external nature regarding policy directions of government and shocks on macroeconomic variables may also moderate the said relationship. Studies in future should therefore control for factors regarding shocks in the economy and effects of policies by government. Furthermore, it would be interesting for further studies to use other approaches in measuring investor sentiment other than the stock returns used in our study to verify its moderating influence in the examined relationship between innovation and performance of domestic Chinese enterprises. Finally, the dataset used for the study consists of state and non-state owned Chinese firms in the quest to examining the link between innovation and corporate performance. Future work should consider analyzing the two categories of firms distinctively so as to ascertain the impact of innovation on the output of each of these categories.

## Data availability statement

Publicly available datasets were analyzed in this study. This data can be found at: ifind database https://www.51ifind.com/.

## Author contributions

CZ: conceptualization and original draft. AI: editing and review and data curation. NE: formal analysis and validation. All authors contributed to the article and approved the submitted version.

## Funding

This study was supported by the Postgraduate Research and Practice Innovation Program of Jiangsu Province (No. KYCX18_2209).

## Conflict of interest

The authors declare that the research was conducted in the absence of any commercial or financial relationships that could be construed as a potential conflict of interest.

## Publisher’s note

All claims expressed in this article are solely those of the authors and do not necessarily represent those of their affiliated organizations, or those of the publisher, the editors and the reviewers. Any product that may be evaluated in this article, or claim that may be made by its manufacturer, is not guaranteed or endorsed by the publisher.
